# Selecting Single Model in Combination Forecasting Based on Cointegration Test and Encompassing Test

**DOI:** 10.1155/2014/621917

**Published:** 2014-04-22

**Authors:** Chuanjin Jiang, Jing Zhang, Fugen Song

**Affiliations:** ^1^Glorious Sun School of Business and Management, Donghua University, Shanghai 200051, China; ^2^Shanghai Business School, Shanghai 200233, China

## Abstract

Combination forecasting takes all characters of each single forecasting method into consideration, and combines them to form a composite, which increases forecasting accuracy. The existing researches on combination forecasting select single model randomly, neglecting the internal characters of the forecasting object. After discussing the function of cointegration test and encompassing test in the selection of single model, supplemented by empirical analysis, the paper gives the single model selection guidance: no more than five suitable single models can be selected from many alternative single models for a certain forecasting target, which increases accuracy and stability.

## 1. Introduction

In 1969, Bates and Granger [1] put forward the idea of “the combination of forecasts” for the first time; that is to say, after the characters of each single forecasting method are considered, combine different single forecasting. This method aroused the attention of forecasting scholars as soon as it was put forward.

The combination forecasting model is the progress of selecting and utilizing the information of single forecasting model, but there are fewer special researches on the selection problem of single model when forming a combination forecasting model. In previous combination forecasting researches and applications, often, there is no screening of single forecasting models involved in combination forecasting, and the forecasting model is chosen according to its obvious characters presented by particular progress and existing knowledge and experience.

During the regression analysis of economic measurement, an important index to evaluate the fitting degree is *R* ^2^, but one of the most direct methods to raise *R* ^2^ is to increase explanatory variables. So a contradiction occurs: after the number of explanatory variables increases, problems such as *t*-tests and colinearity may not be explained. That is to say, it is not the case that the more explanatory variables the better.

The researches of Armstrong [2] prove that the best combinations, most of the time, only use several (no more than 5) methods. When more and more single models are combined, the probability to get the optimum performance decreases.

So, this paper will discuss how to select suitable model from alternative single models to form combination for a certain forecasting target; according to some selecting principles, the number of single models to form combination is no more than five.

## 2. Cointegration Test of Single Model in Combination Forecasting

From the end of the 1980s to the 1990s, a great breakthrough of the modeling theory in econometrics is the cointegration relationship research of the time series. Cointegration actually presents the long-run equilibrium relationship of different time series, which is a key basic thought and theory in the current econometric field and is also an important theoretical cornerstone in current researches on combination forecasting launched by time series.

If forecasting series is an objective performance of sample series, in the long term, they should have an equilibrium relationship, even though, in the short term, these variables may deviate from mean value due to random disturbance and other reasons. Therefore, we can calculate the forecasting series of all alternative single forecasting models and reject the alternative single forecasting models that do not conform to cointegration relationship through the cointegration test with sample series, and the goal of selecting single model can be achieved.

### 2.1. Brief Introduction to Cointegration

Suppose *x*_*t*_ is stationary time series, the mean is *E*(*x*_*t*_), var(*x*_*t*_) is the variance, covariance cov⁡(*x*_*t*_ *x*_*t*+*k*_) is finite, and these numbers can fluctuate according to the changes of time *t*; that is,

(1) $$\begin{array}{c} E\left(x_{t}\right)=E\left(x_{t+h}\right)=\mu ,\\ \text{var}\left(x_{t}\right)=\text{var}\left(x_{t+h}\right)=\sigma^{2},\\ \mathrm{cov}\left(x_{t}x_{t+k}\right)=\mathrm{cov}\left(x_{t+h}x_{t+k+h}\right)=\rho . \end{array}$$

The three mathematical equations above hold true for all *t*, *k*, *h*, and the *μ*, *σ* ^2^, *ρ* here are constants.

Suppose some series requires difference transformation for *d* times before changing into stationary series; this series is integrated of order *d*, denoted by *I*(*d*). Often, economic series is integrated of order one or two.

Suppose *X*_1*t*_, *X*_2*t*_,…, *X*_*kt*_ series are all integrated of order *d* and a vector *α* = (*α*_1_, *α*_2_,…, *α*_*k*_) exists, satisfying

(2) $$\begin{array}{c} Z_{t}=\alpha X_{t}^{\prime }\sim I\left(d-b\right). \end{array}$$

Let *b* > 0,  *X*_*t*_^1^ = (*X*_1*t*_, *X*_2*t*_,…, *X*_*kt*_); it can be considered that (*X*_1*t*_, *X*_2*t*_,…, *X*_*kt*_) is cointegrated of order (*d*, *b*) [3].

The key of this result is to be able to explain that if some linear combination of two variables is *I*(0), they must present the character of tracking each other, and these two variables will not deviate from each other too far in the long term. Therefore, in the long term, one of the necessary conditions for two variables to keep stationary is cointegration.

### 2.2. Cointegration Test of Single Models in Combination Forecasting

Generally speaking, economic series is unstable, so as a relative reasonable forecasting series. Supposing *F*_*t*_ and *Y*_*t*_ are forecasting series and real series, respectively, and both of them are integrated of order *d*, namely, *I*(*d*), only when *F*_*t*_ and *Y*_*t*_ are cointegrated can forecasting error reach *I*(0); otherwise, forecasting error would be unstable; that is, it will augment with time prolonging. The conclusion results in the 2 following theorems.

**Theorem 1**

Given that both predicted series *Y*_*t*_ and forecasting series *F*_*t*_ belong to *I*(*d*) and only when *F*_*t*_ and *Y*_*t*_ are cointegrated can forecasting error reach *I*(0), thus the forecasting error is unstable.

**Theorem 2**

Only when every single forecasting in series *Y*_*t*_'s combination forecasting in *I*(*d*) is cointegrated with *Y*_*t*_ can forecasting error be *I*(0); otherwise, combination forecasting error is unstable.

The former parts in Theorems 1 and 2 are sufficient conditions while the latter parts are necessary conditions. This proposition can be learned that if the combination forecasting or single forecasting in unstable series is in inconformity with the requirements specified in the above proposition, the unstable forecasting error would exist.

Attestation of Theorem 1 can be directly obtained through related definitions of cointegration.

For different combination forecasting models, Theorem 2 has many proven ways, and only the proof process of linear regression method for combination forecasting is given below.

Assuming the forecasting series *Y*_*t*_ is of *I*(*d*) type, then two single forecasting models of *F*_1*t*_ and *F*_2*t*_ in the series are combined to form *C*_*t*_:

(3) $$\begin{array}{c} C_{t}=\beta_{0}+\beta_{1}F_{1t}+\beta_{2}F_{2t}. \end{array}$$

And *β*_1_ + *β*_2_ = 1. Assuming *F*_1*t*_ has a cointegration relation with *Y*_*t*_, *F*_1*t*_ and *Y*_*t*_ are not cointegrated. From the definition of cointegration, it can be known that (*Y*_*t*_ − *F*_1*t*_) belongs to *I*(0), while, for any real number *γ*, (*Y*_*t*_ − *γF*_2*t*_) belongs to *I*(*d*). The error of combination forecasting *C*_*t*_ is recorded into *η*_*t*_; namely,

(4) $$\begin{array}{c} \eta_{t}=-\beta_{0}+\beta_{1}\left(Y_{t}-F_{1t}\right)+\beta_{2}\left(Y_{t}-F_{2t}\right). \end{array}$$

It is obvious in the above equation that *η*_*t*_ belongs to *I*(*d*), meaning that forecasting model has unstable forecasting errors, thus proving Theorem 2, which can easily apply to the combination of *N* kinds of single forecasting methods.

If the single model in combination forecasting is in inconformity with the cointegration conditions in Theorem 2, even creating a combination forecasting model cannot guarantee the forecasting accuracy because the established model forecasting error in this way will be unstable.

### 2.3. Steps of Cointegration Test

Step one of single model cointegration test in combination forecasting is to detect the integration order *d* of predicted series and the integration order *d*′ of single forecasting series as Theorem 1. If the single forecasting series is cointegrated with predicted series, then *d* = *d*′. When the predicted sample series has the same integration order *d* as single forecasting series, the cointegration test can be made to the predicted sample series and single forecasting series, and the specific steps are as follows.

#### 2.3.1. Integration Order Testing

The most widely used method is unit root test for integration order calculation of sample series or forecasting series. Standard unit root test method is Dicker-Fuller testing (DF), but the frequently used method in reality is generalized DF testing (ADF) [4]. ADF testing is actually making the following regression:

(5) $$\begin{array}{c} \Delta x_{t}=\beta_{1}x_{t-1}+\beta_{2}\Delta x_{t-1}+\beta_{3}\Delta x_{t-2}+\beta_{4}+\beta_{5}t. \end{array}$$

The above formula adopts the lagged term *β*_1_ *x*_*t*−1_ of first degree and two differential lagged terms *β*_2_Δ*x*_*t*−1_ and *β*_3_Δ*x*_*t*−2_ of time series with the purpose to transform residue term to the white noise, and the number of terms is determined according to actual situations; constant term *β*_4_ and time trend term *β*_5_ *t* represent the first difference of time series.

Unit root test is mainly performed to test the coefficient *β*_2_ of the regression expression *x*_*t*−1_; if the coefficient significance was unequal to 0, the hypothesis that *x* has the unit root is rejected and *x* is a smooth series. Then the following hypothesis testing can be made:

(6) $$\begin{array}{c} H_{0}\text{:}\;\;\beta_{2}=0,\;\;H_{1}\text{:}\;\;\beta_{2}<0. \end{array}$$

The output result of ADF test includes the statistical magnitude *t* of coefficient *β*_2_ in differential lagged terms and the tested critical value of the assumption whether it equals 0. If the output result is a negative number far smaller than 0, then *H*_1_ is accepted and *H*_0_ is rejected, which also indicates that the time series is stable with no unit root. The stability of first difference is also needed to be tested for unstable time series. If stable, then the series can be deemed as *I*(1) type. If unstable, the first difference will continue the difference till the series becomes stable; when the series becomes stable after *n*-time differences, the integration order of the series is *n*.

#### 2.3.2. Cointegration Test

To know whether two variables *X* and *Y* have cointegration correlation, EG two-step method [5] can be used for verification.

*Step  1*. Evaluate the following equation by ordinary least squares (OLS):

(7) $$\begin{array}{c} Y_{t}=\alpha_{t}X_{t}+\varepsilon_{t}. \end{array}$$

So it can lead to

(8) $$\begin{array}{c} {\hat{Y}}_{t}={\hat{\alpha }}_{t}X_{t}. \end{array}$$

It is called cointegration regression.

*Step  2*. Test the integrity of ${\hat{e}}_{t}$. If it is a stable series, then variables *X*, *Y* can be considered as cointegrated of order (1,1); if ${\hat{e}}_{t}$ is integrated of order, then variables *X*, *Y* can be considered as cointegrated of order (1, 2).

The testing methods for ${\hat{e}}_{t}$'s integrity are the above DF testing or ADF testing.

Of course, in the specific application process, the following manner can also be carried out.

Expand the ADF regression of *y*_*t*_ to *x*_*t*_ through OLS method:

(9) $$\begin{array}{c} y_{t}=\text{const}+\beta x_{t}+\varepsilon_{t}. \end{array}$$

Study the stability of the obtained *ε*_*t*_ regression residuals from this equation; if stable, then it indicates the presence of cointegration process, the equation can also be called cointegration equation, and the equation expresses the cointegration relationship between variables. The stability of the obtained *ε*_*t*_ regression residuals can be tested by ADF testing method; that is, it makes the following regression:

(10) $$\begin{array}{c} \Delta u_{t}=\gamma_{0}u_{t-1}+\overset{k}{\underset{i=1}{\sum }}\gamma_{i}\Delta u_{t-i}+v_{t}. \end{array}$$

In formula (10), the new error term is *v*_*t*_; *k* is the optimal lagged order to transform residue term *v*_*t*_ to white noise. Lagrange multiplication testing method or Durbin-Watson (DW) testing can be employed to determine whether the residual terms are white noise.

*t* testing in ${\hat{\gamma }}_{0}$ is ADF statistics; if it exceeds the critical value when the significance level is 0.01, 0.05, or 0.10, then the original hypothesis of noncointegration is rejected.

### 2.4. Empirical Analysis

M3-Competition sample series [6] is used below for empirical analysis of single model cointegration validation. The paper selects the 999th series in M3 (denoted by N999) as verification series; N999 is a macroeconomic series and quarterly data with totally 52 sample points, of which the first 44 samples are fitting samples while the last 8 are forecasting samples, (see Tables 1 and 2).

In the paper, 12 single models are selected to fit and predict N999 series, which are, respectively, moving average (Naïve), single exponential smoothing (Single), linear exponential smoothing (Holts), dampen trend exponential smoothing (Dampen), seasonal exponential smoothing (Winter), time series decomposition model (Decomposition), ARIMA, ARARMA, BP neural network (BP), NARX neural network (NARX), grey forecasting (GM), and support vector machine (SVM). Real things contain two kinds of information: the nonlinear information and linear information; therefore, some classical linear and nonlinear single models are considered in the paper.

The evaluation indexes of forecasting are of a large number, and Goodwin and Lawton [7] have listed many indicators used in forecasting evaluation currently; the paper selects “symmetric mean absolute percentage error” as the forecasting evaluation index. *Y*_*t*_ is the observation value at time *t*, and *F*_*t*_ represents the forecasting value of *Y*_*t*_; then

(11) $$\begin{array}{c} \text{sMAPE}=\frac{1}{N}\overset{N}{\underset{t=1}{\sum }}\left|\frac{\left(Y_{t}-F_{t}\right)}{\left(Y_{t}+F_{t}\right)}\right|\times 200. \end{array}$$

sMAPE is proposed for the greater punishment shortcomings of MAPE to positive error. For example, if *Y*_*t*_ = 100, *F*_*t*_ = 50, then MAPE = 50; if *Y*_*t*_ = 50, *F*_*t*_ = 100, then MAPE = 100. But in both cases, sMAPE are 66.7. Furthermore, fluctuation range of sMAPE is 0–200, while MAPE has no upper limit.

Next, we conduct unit root test to N999 sample series through the test tool of Eviews6.0 software.

It can be observed from Table 3 that after a first difference to N999 sample series, ADF statistic magnitude is −5.025, less than the 1% critical level. Therefore, N999 sample series can be determined as integrated of order; namely, it belongs to *I*(1).

Next, unit root test is conducted for the fitting data of twelve single models, and only when the fitting sample of single model is also integrated of order can they go into cointegration test phase (see Table 4).

Through the unit root test, we can see that the Naïve and Single are stable series, and the integrated order of Auto-ANN is greater than 1, so these three single models are removed first.

The cointegration test is conducted to fitting series and sample series of the remaining nine single models; according to (9) and (10), stability test is actually made to the residues of nine single models.  Table 5 shows the ADF testing of fitting sample residues of nine single forecasting models.

It can be observed from Table 5 that the forecasting of DAMPEN and GM(1,1) to fitting sample residue series of two single models does not pass ADF testing, so it is unstable; therefore these two single forecasting models do not pass the cointegration verification.

Finally, the remaining seven single models are predicted through the combination of simple average, weighted optimal, and ANN forecasting model again, and the result is shown in Table 6.

It can be observed from Table 6 that the combination forecasting accuracy after single model cointegration screening has improved to a certain extent, though not obvious. More importantly, forecasting accuracy of simple average combination (4.90) exceeds the Naïve (4.91) with little advantage, but it at least proves the cointegration verification validity of single forecasting model.

## 3. Encompassing Test of Single Model in Combination Forecasting 

Numerous research literatures on forecasting accuracy mostly focus on the forecasting accuracy and stability evaluation and cannot directly compare the differences between competing forecastings of the same object. To achieve such discriminative comparison, forecasting encompassing theory came naturally, which was proposed by Meese and Rogoff [8, 9]: if a forecasting model contains or includes forecasting model B-related information, we believe that model A includes model B.

### 3.1. Significance of Encompassing Test of Single Model in Combination Forecasting

Kisinbay [10] establishes a statistical magnitude of approximate pivot in line with *t* distribution to apply encompassing test to single model selection in combination forecasting so as to filter the included single models and reduce the single model quantity involved in the combination. He believes that adding the included single models into combination models has not any sense and even increases the complexity of the algorithm.

Tomiyuki and Ryoji [11] point out that finding the single models of high predictability and small correlation degree with other models is important to improve the combination forecasting effects. Encompassing test is the answer to the competing models in combination forecasting. Therefore, it seems extremely important to create encompassing methods and principles of combination forecasting on the basis of statistical approaches, so as to improve combination forecasting efficiency.

Encompassing test can identify the origins of accuracy differences between competing models; help predictors distinguish whether the difference is caused by sample variability or the significance of information set in construction models. But we cannot ensure that the forecasting model with the best forecasting accuracy performance must be able to explain all competing models. The research of Ericsson [12] shows that taking advantages in forecasting accuracy out of samples is not a sufficient condition for forecasting encompassing. In our empirical analysis, we further find that when the forecasting model A includes model B, the model A's accuracy may be lower than B's for containing more useless or inferior information.

Combination forecasting theory holds that the information in different forecasting models may vary due to the differences of models, even leading to different forecasting results. We can obtain better results than the forecasting values of originally two single models through combination forecasting. However, if no additional information is included in the constructed combination forecasting, this combination will be not able to get higher efficiency, and thus the combination forecasting loses its significance.

Based on the above analysis, besides cointegration test to single models before combination forecasting, we should also adopt forecasting encompassing test to identify the containment relationships between alternative single models to select single modes through certain heuristic strategies.

### 3.2. Basic Principle of Encompassing Test in Single Model Selection for Combination Forecasting

We first consider the two-two encompassing between single forecasting models by making *f*_1_^(*t*)^ *f*_2_^(*t*)^ the forecasting values of two single forecasting models at time *t*, and they have the following regression equation:

(12) $$\begin{array}{c} y_{t}=\beta_{1}f_{1}^{\left(t\right)}+\beta_{2}f_{2}^{\left(t\right)}+\varepsilon_{t}. \end{array}$$

That model *f*_1_^(*t*)^ encompasses model *f*_2_^(*t*)^ can be written as (*β*_1_, *β*_2_) = (1,0), and in turn, it can also be that model *f*_2_^(*t*)^ includes model *f*_1_^(*t*)^. If (*β*_1_, *β*_2_) has other values, then the two models are intolerant and each model contains independent *y*_*t*_-related information. If the covariance is relatively stable, then some standard methods can be used for encompassing verification test. If no any predictive model includes other models, it can be shown that all models are wrong (i.e., no one model can reflect the true data generating process); and only by relying on the combination forecasting can combination forecasting be made to useful data information.

Ericsson [12] constructs an encompassing test model:

(13) $$\begin{array}{c} y_{t}=\beta_{1}f_{1}^{\left(t-s,t\right)}+\beta_{2}f_{2}^{\left(t-s,t\right)}+\varepsilon_{t}. \end{array}$$

*f*_1_^(*t*−*s*,*t*)^ means the forecasting value of the information at time *t* − *s* used by *f*_1_^(*t*)^ to *y*_*t*_, and *f*_2_^(*t*−*s*,*t*)^ means the forecasting value of the information at time *t* − *s* used by *f*_2_^(*t*)^ to *y*_*t*_. Under the restriction condition that *β*_1_ + *β*_2_ = 1, it is tested that *β*_1_ = 1 or *β*_2_ = 0.

Fair and Shiller [13] propose the following encompassing test model:

(14) $$\begin{array}{c} \Delta_{s}y_{t}=a_{0}+\beta_{1}\left(f_{1}^{\left(t-s,t\right)}-y_{t-s}\right)+\beta_{2}\left(f_{2}^{\left(t-s,t\right)}-y_{t-s}\right)+\varepsilon_{t}. \end{array}$$

If Δ_*s*_ *y*_*t*_ = *y*_*t*_ − *y*_*t*−*s*_, *β*_1_ = 0, and *β*_2_ ≠ 0, model *f*_1_^(*t*)^ is included by model *f*_2_^(*t*)^; otherwise, model *f*_2_^(*t*)^ is included by model *f*_1_^(*t*)^, and *β*_1_ and *β*_2_ are unconstrained.

In testing process through (14), if the two models have the same information, the forecasting results will show a high correlation degree, and thus *β*_1_ and *β*_2_ are difficult to distinguish. Although this problem can obtain the evaluation of (14) through 0 regressions, the test results are not obvious.

Harvey, Leybourne, and Newbold provide HLN encompassing testing method for the above model defects [14]. HLN testing can verify and test whether two competing models have similar performances, and considering one simple *c*_*t*_ of loss difference sequence, it has

(15) $$\begin{array}{c} c_{t}=L\left(e_{1t}\right)-L\left(e_{2t}\right), \end{array}$$

where *L*( ) represents any loss function, such as sMAPE or MSE; *e*_1*t*_ is the step forward forecasting error of model *i* to *t*; *i* = 1,2; *t* = 1,…, *T*. And the forecasting performance shows that the expected value of *c*_*t*_ is 0, namely, *E*(*c*_*t*_) = 0; besides, the test adopts the mean of observation sample *c*_*t*_. Assumed that the loss difference sequence has the stability of covariance, then, under the null hypothesis of the same forecasting accuracy, the test statistical magnitude of HLN will also be more consistent with normal distribution, and the specific formula of the statistical test is as follows:

(16) $$\begin{array}{c} \text{HLN}=\frac{\bar{c}}{\sqrt{\hat{V}\left(\bar{c}\right)}}. \end{array}$$

Assuming that the *t* step forward forecasting depends on the ordinal number *t* − 1 and $\hat{V}\left(\bar{c}\right)$ is the consistent evaluation of asymptotic variance $\bar{c}$, we can calculate $\hat{V}\left(\bar{c}\right)$ through the following formula:

(17) $$\begin{array}{c} \hat{V}\left(\bar{c}\right)\approx \frac{1}{T}\left(\gamma_{0}+2\overset{\tau -1}{\underset{i=1}{\sum }}\gamma_{i}\right). \end{array}$$

Here *γ*_*i*_ is the *i*th autocovariance of $\bar{c}$, whose size can be estimated by ${\hat{r}}_{i}=T^{-1}\sum_{t=i+1}^{T}\left(c_{t}-\overset{-}{c}\right)\left(c_{t-i}-\overset{-}{c}\right)$.

Through the Monte Carlo method, we simulate and evaluate the testing capacity of HLN and find that it has poor performance in dealing with small samples.

Therefore, the paper amends on HLN testing so that its performance in testing small samples improves (after amendments, it can be referred to as MHLN testing). Specific amendments are as follows: first, compare and test statistical magnitude through *t* distribution critical value of *T* − 1 degree of freedom, rather than a normal distribution; second, modify test statistical magnitude as follows:

(18) $$\begin{array}{c} \text{MHLN}=T^{-1/2}{\left[T+1-2t+T^{-1}t\left(t-1\right)\right]}^{1/2}\text{HLN}. \end{array}$$

Testing for MHLN statistic magnitude is very simple, and only by calling correlation test function in Matlab7.0 statistical toolbox can test results of various significant levels be obtained.

### 3.3. Steps of Encompassing Test of Single Models in Combination Forecasting

This section chooses N999 series in M3 as the sample series of encompassing test; through the cointegration selection in Section 2.2, the original twelve single forecasting models are only chosen to seven: Holts linear exponential smoothing, WINTER seasonal exponential smoothing, THETA time series decomposition model, BJ auto ARIMA, ARARMA, NARX neural networks, and SVM support vector machine. Here we will use MHLN encompassing testing described above for N999 sample series to conduct encompassing testing for the alternative 7 single models.

**Step 1**

Calculate the fitting sMAPE in the samples of 7 single models for N999 series and sort all models according to that numerical number and the performance of each model. It should be noted that the fitting sMAPE in the samples of single models is different from the forecasting sMAPE out of samples.

**Step 2**

Select the model with the lowest sMAPE value as the optimal model, and for optimal model NARX neural network, the adopted MHLN encompassing test (confidence level of 0.01) and *t* distribution table show that the remaining six single models are all not included by the optimal model.

**Step 3**

Select the second optimal model ARARMA and operate as step two, and the result shows that ARIMA is included by ARARMA, so we remove the ARIMA model among alternative single models.

**Step 4**

Select the third optimal model of Decomposition and operate as step two again until the end of encompassing test and no included single forecasting model is found.

Since the number of single models in combination forecasting is better to be no more than 5, we repeat the above encompassing test procedures again and increase confidence level to 0.05. And the result shows that the Decomposition includes SVM.

Through encompassing test, seven single models are finally selected into five models. These five single models are used to make combination forecasting again, and further analysis is made on the basis of Table 6. For comparison, six single models that are selected the first time are compared together (confidence level of 0.01) (see Table 7).

We find that ARARMA includes ARIMA; but in fact, the real forecasting accuracy out of ARIMA samples is higher than that of ARARMA, which also proves that “taking advantages in forecasting accuracy out of samples is not a sufficient condition for forecasting encompassing” mentioned at the beginning of this chapter.

Furthermore, we find that when confidence level is 0.01, the simple average combination accuracy of the remaining six single models in conclusiveness test decreases while the accuracy of the weighted optimal combination upgrades. The main reason is that the eliminated ARIMA has a high forecasting accuracy itself, whose exclusion will necessarily decrease the accuracy of simple average combination; but for weighted optimal combination, the exclusion of ARIMA leads to the significant increase of weights of ARARMA and NARX neural network, so the combination forecasting accuracy improves.

When the confidence level is 0.05, SVM is excluded; the accuracy of the remaining five single model combinations has significantly improved. In particular, the sMAPE value of weighted optimal combination is lower than the Naïve single forecasting accuracy, proving the effectiveness of combination forecasting.

Therefore, when the single models are under encompassing tests, if the remaining single model number is no more than 5 when under the test at confidence level of 0.01, the test directly ends; if greater than 5, then confidence level should be adjusted to 0.05 or 0.1.

## 4. Conclusion

This thesis discusses single forecasting model selection in combination forecasting through cointegration test first and encompassing test method then. The result shows that the forecasting accuracy has improved to a certain extent after single model selection.

Cointegration verification aims to keep the fitting sample and real sample of single models have a consistent fluctuation trend, thus guaranteeing the combination forecasting accuracy of samples to the largest extent, especially the accuracy of middle and long-term forecasting. And the encompassing test is to eliminate the inclusive models; the start point of single model forecasting lies in the fact that each model establishes on different information set and owns different model type. If one single model is included by another single model, it will lose its meaning in the combination model and only increase the burden of combination.

According to the empirical analysis about N999 sample series, the paper screens 12 alternative single models to the final 5; and finally the forecasting accuracy is calculated to prove the efficiency of single model selection.

## Figures and Tables

**Table 1:** N999 fitting sample series.

Quarter	Year										
	1990	1991	1992	1993	1994	1995	1996	1997	1998	1999	2000
1	3944.5	3969	4389	4244	4497	4938.5	5031	4895	5120.5	5790	6302.5
2	3901	4068	4311.5	4244	4475.5	5012.5	4994	5034	5262.5	5944.5	6207
3	3802.5	4250	4274.5	4185	4697.5	5071	4984.5	5018.5	5355	5984.5	6805.5
4	3737.5	4373.5	4222	4370.5	4913.5	4978.5	4975.5	5145	5475.5	6009.5	7185

**Table 2:** N999 forecasting sample series.

Quarter	Year	
	2001	2002
1	7373.5	7608
2	7259.5	7592.5
3	7558.5	7722
4	7660.5	7611

**Table:** (a)

	*t*-statistic	Prob.*
Augmented Dickey-Fuller test statistic	−5.025046	0.0002

Test critical values: 1% level	−3.596616	
5% level	−2.933158	
10% level	−2.604867	

*MacKinnon (1996) one-sided *P* values.

**Table:** (b)

Augmented Dickey-Fuller test equation				
Dependent variable: *D*(*M*, 2)				
Method: least squares				
Date: 09/25/13, time: 00:18				
Sample (adjusted): 1990Q3 2000Q4				
Included observations: 42 after adjustments				

Variable	Coefficient	Std. error	*t*-statistic	Prob.

*D*(*M*(−1))	−0.822358	0.163652	−5.025046	0.0000
*C*	66.08964	24.93664	2.650303	0.0115

*R*-squared	0.386983	Mean dependent var.		10.07143
Adjusted *R*-squared	0.371658	S.D. dependent var.		182.3687
S.E. of regression	144.5601	Akaike info. criterion		12.83172
Sum squared resid.	835905.0	Schwarz criterion		12.91446
Log likelihood	−267.4660	Hannan-Quinn criter.		12.86205
*F*-statistic	25.25109	Durbin-Watson stat.		2.023140
Prob. (*F*-statistic)	0.000011			

**Table 4:** Unit root test on fitting data of 12 single forecasts.

Test sample	ADF statistics	1% critical value	IS stable	ADF statistics after first difference	1% critical value	IS stable after first difference	Result
Forecast sample	2.66	−3.59	No	−5.03	−3.60	Yes	*I*(1)
Naive	−6.02	−3.60	Yes	—	—	—	*I*(0)
SINGLE	−6.02	−3.60	Yes	—	—	—	*I*(0)
Holts	1.65	−3.60	No	−7.53	−3.59	Yes	*I*(1)
DAMPEN	0.15	−3.60	No	−16.02	−3.61	Yes	*I*(1)
WINTE	4.20	−3.59	No	−15.31	−3.61	Yes	*I*(1)
THETA	5.12	−3.59	No	−9.01	−3.60	Yes	*I*(1)
ARIMA	3.31	−3.60	No	−7.59	−3.59	Yes	*I*(1)
ARARMA	2.08	−3.60	No	−11.4	−3.60	Yes	*I*(1)
Auto-ANN	10.8	−3.61	No	1.55	−3.59	No	*I*(2) or more
NARX	5.21	−3.60	No	−19.74	−3.60	Yes	*I*(1)
GM(1, 1)	3.11	−3.59	No	−12.56	−3.59	Yes	*I*(1)
SVM	4.10	−3.60	No	−4.21	−3.60	Yes	*I*(1)

**Table 5:** ADF test on residuals of 9 single forecasts' fitting sample.

The single forecast model residual sequence	ADF statistics	1% critical value	IS stable	IS cointegrated with sample series
Holts	−5.47	−4.21	Yes	Yes
DAMPEN	−2.56	−4.20	No	No
WINTER	−7.12	−4.22	Yes	Yes
THETA	−7.33	−4.21	Yes	Yes
ARIMA	−9.35	−4.23	Yes	Yes
ARARMA	−12.38	−4.22	Yes	Yes
NARX	−15.21	−4.23	Yes	Yes
GM(1, 1)	1.21	−4.22	No	No
SVM	−4.89	−4.21	Yes	Yes

**Table 6:** Result of combined forecasts of single models by cointegration test.

	Simple average	Weighted optimal	ANN
12 single models' combination without cointegration test	5.00	5.42	5.88
7 single models' combination with cointegration test	4.90	5.21	5.44

**Table 7:** sMAPE of combined forecast of single models by inclusive test.

	Simple average	Weighted optimal	ANN
12 single models' combination without cointegration test	5.00	5.42	5.88
7 single models' combination with cointegration test	4.90	5.21	5.44
6 single models' combination with encompassing test (0.01 confidence level)	5.31	5.14	5.69
5 single models' combination with encompassing test (0.05 confidence level)	4.62	4.88	5.32
